# Diversity of Antimicrobial Peptides in Silkworm

**DOI:** 10.3390/life13051161

**Published:** 2023-05-11

**Authors:** Pooja Makwana, Kamidi Rahul, Katsuhiko Ito, Bindu Subhadra

**Affiliations:** 1 Central Sericultural Research & Training Institute, Central Silk Board, Ministry of Textiles, Government of India, Berhampore, Murshidabad 742101, West Bengal, India; pooja.makwana@nic.in (P.M.);; 2 Laboratory of Sericultural Science, Department of Science of Biological Production, Graduate School of Agriculture, Tokyo University of Agriculture and Technology, 3-5-8 Saiwai-cho, Fuchu-shi 183-8509, Tokyo, Japan; 3 Department of Veterinary Biomedical Sciences, College of Veterinary Medicine, Long Island University, Brookville, New York, NY 11548, USA

**Keywords:** antimicrobial resistance, silkworm, innate immunity, antimicrobial peptides, novel therapeutics

## Abstract

Antimicrobial resistance is a phenomenon that the present-day world is witnessing that poses a serious threat to global health. The decline in the development of novel therapeutics over the last couple of decades has exacerbated the situation further. In this scenario, the pursuit of new alternative therapeutics to commonly used antibiotics has gained predominance amongst researchers across the world. Antimicrobial peptides (AMPs) from natural sources have drawn significant interest in the recent years as promising pharmacological substitutes over the conventional antibiotics. The most notable advantage of AMPs is that microorganisms cannot develop resistance to them. Insects represent one of the potential sources of AMPs, which are synthesized as part of an innate immune defence against invading pathogens. AMPs from different insects have been extensively studied, and silkworm is one of them. Diverse classes of AMPs (including attacins, cecropins, defensins, enbocins, gloverins, lebocins and moricins) were identified from silkworm that exhibit antimicrobial property against bacteria, fungi and viruses, indicating their potential therapeutic benefits. This review briefs about the immune responses of silkworm to invading pathogens, the isolation of AMPs from silkworms, AMPs reported in silkworms and their activity against various microorganisms.

## 1. Introduction

Antimicrobial resistance is considered to be one of the major threats to global public health. This menace has emerged in the recent decades as a result of antibiotic misuse in human and animal healthcare, particularly in most of the developing nations [1,2,3]. Antimicrobial resistance is a phenomenon that arises when microbes such as bacteria, fungi, parasites and viruses develop resistance to drugs to which they were previously susceptible, making infections more difficult to treat. This is a serious issue because a resistant infection has the potential to be fatal, contagious and extremely expensive for both individuals as well as society (URL—https://www.who.int/india/antimicrobial-resistance: accessed on 27 February 2023). By the year 2050, infections resistant to antibiotics are anticipated to result in the deaths of up to ten million people annually and are likely to cost the world economy over $100 trillion. Considering the gravity of the situation, the World Health Organization listed antimicrobial resistance as one of the top ten health threats being faced by humanity worldwide [4,5].

Furthermore, adding to the difficulty of this endeavour, the development of new classes of antimicrobial agents has declined over the past thirty-five years. This might be attributed to the long-standing conventional approaches used in the search for antibiotics or perhaps the fact that many of the natural structures that exhibit antimicrobial action have already been identified [6,7,8]. As a result, there is a significant amount of interest in discovering novel antibiotic classes with therapeutic potential for treating various infectious diseases in both humans and animals.

In recent years, there has been a tremendous increase in the screening of natural products for the development of novel therapeutics. Natural compounds exhibit vast chemical diversity, making them important and reliable sources of novel medications [9]. AMPs are one such group of natural products produced by a wide range of organisms in response to pathogenic stimuli and are important components of an innate immune system [10]. Ever since the discovery of an antimicrobial compound, gramicidin from a bacterial strain belonging to the genus *Bacillus* by Rene Dubos in 1939, a number of AMPs have been isolated and characterized [3,11]. Approximately 3500 AMPs from a variety of organisms, including birds, cattle, fish, frogs, humans, insects, microbes, plants and reptiles, were registered in the antimicrobial peptide database [12] (URL—https://aps.unmc.edu/home: accessed on 27 February 2023). According to a research report dated in 2020, seven AMPs received approval from the U.S. Food and Drug Administration for usage and are commercially available [7].

AMPs are typically described as polypeptide antimicrobial compounds with fewer than one hundred amino acid residues that are encoded by genes and synthesized by ribosomes [13]. In general, the majority of the AMPs are cationic and are well known for their ability to preferentially interact with phospholipid bilayers of bacterial cell membrane. Furthermore, AMPs typically contain nearly 50% hydrophobic residues. As a result, AMPs display spatially divided hydrophilic as well as hydrophobic moieties and show amphipathic characteristics upon interaction with membranes. AMPs’ activities are generally ascertained by their interactions with cell membranes of bacteria. AMPs initially bind to the lipopolysaccharides of Gram negative bacteria or the lipoteichoic acids of Gram positive bacteria through electrostatic interactions, after which bacterial cell membranes will be permeabilized and disrupted, leading to cell death [14,15]. Unlike conventional antimicrobial medications, most of the AMPs bind to the cell membranes of bacteria and do not rely upon the presence of specific receptors, making them ideal for combating resistance caused by bacterial mutations [16]. The majority of the AMPs inhibit microbial (bacterial) growth via membrane interactions, whereas a few of them are also reported to arrest growth by interfering with protein synthesis, nucleic acid synthesis, cell division or protease activity [17]. Despite the various mechanisms depicted above, it must be noted that the pathways underlying the antimicrobial action of AMPs are not fully understood [18]. Apart from antibacterial properties, AMPs have also been shown to be effective against a wide range of microbes including fungi, viruses and parasites [19,20]. The majority of the AMPs discovered to date are antibacterial peptides followed by antifungal, antiviral and antiparasitic peptides [3].

Insects represent one of the most abundant organisms inhabiting earth and contribute to numerous ecosystem services [21]. With the developments in insect biotechnology also termed as yellow biotechnology, insects are being successfully explored for a wide range of bioactive compounds that are currently in use across diverse sectors [22]. One such bioactive molecule derived from insects is AMPs. The first AMP from insects was extracted from the pupae of *Hyalophora cecropia* and, since then, numerous insect-derived AMPs have been discovered to date [18,23]. The accessible information with respect to insect genomes as well as transcriptomes coupled with the possibility to directly analyse insects’ haemolymph samples using proteomic approaches has led to the identification of numerous novel AMPs in the recent years. AMPs from insects are generally categorized based on their structural or functional properties. The three main structural groups include linear α-helical peptides devoid of cysteine moieties (cecropin, moricin), peptides with a β-sheet globular structure (defensins) and peptides encompassing high numbers of particular amino acids like proline (lebocin) or glycine (attacin, gloverin) [24]. The functional classification of insect AMPs largely depends on the target pathogen and is not based on mechanism of action. Attacins, cecropins, defensins and AMPs rich in proline constitute the vast majority of insect AMPs and have been discovered in more than a couple of insect orders, although moricin and gloverin have only been reported in lepidopteran insects [23].

Insect-derived AMPs are reported to inhibit the growth of bacterial pathogens belonging to various genera. Prominent among them are multiple drug resistant (MDR) bacteria including *Escherichia coli*, *Klebsiella pneumoniae*, *Streptococcus sanguinis* and *Staphylococcus aureus*. Few AMPs from insects also exhibited antiviral activity against human influenza viruses A and B and herpes simplex virus 1. Diverse fungal strains (*Aspergillus* sp., *Botrytis* sp., *Cryptococcus* sp., *Fusarium* sp.) were also reported to be susceptible to AMPs from insects [2].

AMPs are the most sought after therapeutic compounds due to their characteristics, which include low toxicity to humans and animals; high specificity and improved efficacy against target microbes when compared to conventional antibiotics; and, most importantly, the fact that majority of microbes cannot develop resistance to AMPs. Most of the AMPs disrupt the bacterial cells via nonspecific interactions with their membrane surface. AMPs attack numerous low affinity targets such as bacterial membranes, as opposed to traditional antibiotics, which act through a specific high-affinity antimicrobial target and can result in microbial resistance. The swift microbicidal property of AMPs is also considered as a factor that prevents evolution of resistance. These might be the most probable reasons for minimal/restricted emergence of bacterial resistance against most of the AMPs [12,25]. However, there are reports suggesting bacteria can evade the action of AMPs by a myriad of strategies. This mostly involve structural modifications in the cell wall/membrane including D-alanylation (incorporation of D-alanine in the lipoteichoic acids) resulting in a decrease in negative membrane charge; lysinylation (adding of L-lysine to phosphatidylglycerol); O-acetylation/N-deacetylation of the peptidoglycan and glycosylation of the cell wall teichoic acids [26]. Lipopolysaccharide modifications induced by adding phosphoethanolamine or 4-amino-4-deoxy-L-arabinose to the core and lipid-A portions, acetylation of the O-antigen and fatty acid hydroxylation are the most common mechanisms of resistance to AMPs reported in Gram negative bacteria [27]. A few more strategies employed by microbes to gain resistance against AMPs include capsule production, biofilm formation, expulsion of AMPs by efflux pumps, secreting specific proteases that cleave AMPs, signalling mechanisms that result in expression of genes conferring resistance to AMPs and regulation of hosts AMP gene expression [26,27].

AMPs, apart from being used in medicine and allied fields, also find their application in various sectors viz., food processing, animal husbandry, aquaculture and agriculture [28]. Besides the many advantages as mentioned above, AMPs also have a few disadvantages. A few AMPs may elicit cytotoxicity, limiting their use in therapeutic applications. However, reports of AMPs from diverse sources/modified AMPs possessing broad spectrum antibacterial activity with negligible or low cytotoxicity including Ll14 [29], Lys-linked homodimers of buforin II [30], PEP-1 [31], Melectin [32], DRS-CA-1 and DRS-DU-1 [33], Cp1 [34], helix-PXXP-helix peptide [35] and Citropin 1.1 and Temporin A [36] are also reported. AMPs are reported to be susceptible to proteolytic degradation, affected by pH and ionic strength, and exhibit low stability at ambient temperatures. The costs involved in the synthesis of AMPs are also high and there are not many commercial manufacturers that produce the same, further limiting their use [12].

## 2. Silkworm and Immune Responses

**Figure 1:**
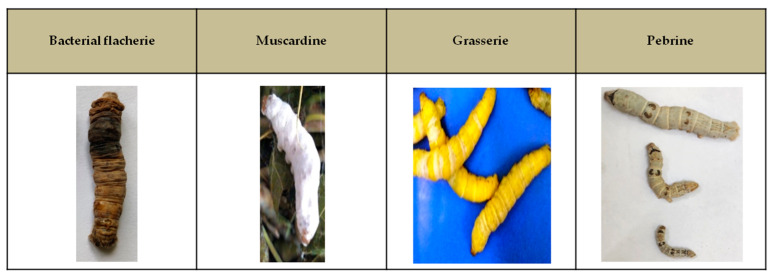
Infected larvae showing the symptoms of different silkworm diseases.

The silkworm *Bombyx mori* has long been utilized to produce silk and is economically significant in many countries that practice sericulture. Silkworms are susceptible to infection by a variety of microbial pathogens including bacteria, fungi and viruses. Apart from microbial pathogens, silkworms are also vulnerable to pests such as *Exorista bombycis* that infects the larval stage and *Dermestid aeter* that attacks pupal or moth stages. The causal microorganisms as well as specific symptoms of different silkworm diseases and the images of infected silkworms are depicted in Table 1 and Figure 1, respectively.

**Figure 2:**
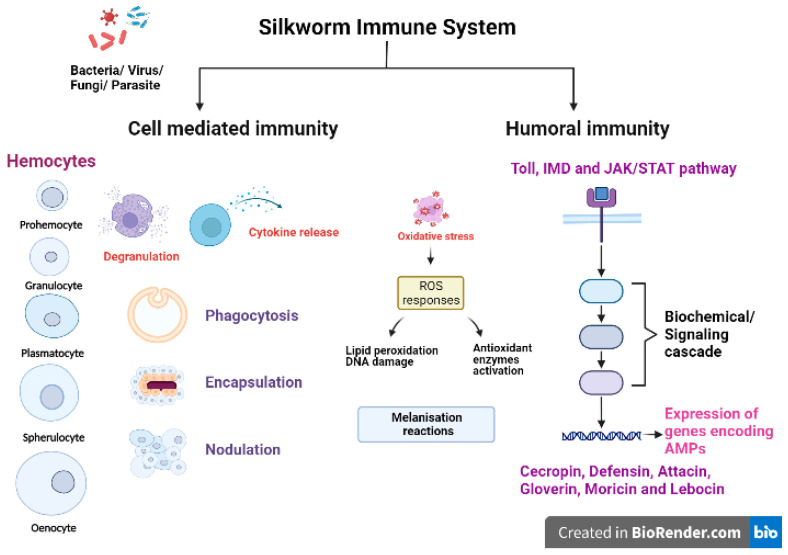
Innate immune mechanisms in silkworm (Created in BioRender.com).

Like any other insect, silkworms lack an adaptive immune mechanism and solely depend on their innate immune system to combat the invading pathogenic microorganisms/pests [37]. The silkworm *B. mori* has an efficient innate immune system to fight against various pathogens and pests. The first line of defence against pathogenic infection in silkworms is provided through physical barriers such as integument (a protective exoskeleton) and the peritrophic matrix, a semipermeable membrane layer that protects the midgut epithelium [38,39]. Upon crossing the physical barriers, the innate immunity of the silkworms is activated, which is comprised of cellular and humoral immune reactions (Figure 2). The first step of host immune responses is the recognition of pathogen-associated molecular patterns (PAMPs) of pathogen or parasite as nonself by pattern recognition receptors (PRRs) allowing the host to react against invading organisms and initiate further immune responses. The PAMPs include peptidoglycan (PGN), lipopolysaccharides (LPS) and β-1,3-glucan present in cell wall of bacteria and fungi [40,41,42]. The host PRRs are germline encoded proteins such as lectins, C-type lectins, PGN recognition proteins (PGRPs), β-1,3-glucan recognition receptors (βGRPs), haemolin, haemocytin, G-binding proteins (GNBPs) and Toll-like receptors (TLRs) [43,44]. The βGRPs/Gram negative bacteria binding protein 3 (GNBP3) bind to β-1,3-glucan in the fungal cell wall to activate the pro-phenoloxidase cascade and Toll signalling pathway in silkworms [40]. *Bm*PGRP2-1 (a transmembrane protein) and *Bm*PGRP2-2 (an intracellular protein) are two important PGRPs in *B. mori* that activate the immunodeficiency (Imd) pathway and suppress the PTEN-phosphoinositide 3-kinase (PI3K)/Akt signalling, respectively, in response to *B. mori* nucleopolyhedrovirus (*Bm*NPV) infections [43].

Cellular immunity is mainly carried out by haemocytes (free circulating immunosurveillance cells), which are classified on the basis of morphology and size into five types: prohemocytes (Pr), granulocytes (Gr), plasmatocytes (Pl), spehrulocytes (Sp) and oenocytes (Oe) [41,45,46,47]. Prohemocytes are progenitor cells mainly found in hematopoietic organs (HPOs) in insects, and these cells differentiate into other types of haemocytes [48]. Plasmatocytes and granulocytes play major role in the recognition and activation of immune responses due to their capability to adhere to foreign surfaces [47]. The oenocytes are mainly engaged in melanisation reactions as they are rich in prophenoloxidases (PPO) while spherulocytes function in silkworms is largely unknown. Haemocytes exert aggregation, granulation, degranulation and melanisation reactions to cope up with infections and initiate cellular responses [49,50,51]. The major cell-mediated immune reactions exhibited by haemocytes include phagocytosis, nodulation and encapsulation reactions [52]. Plasmatocytes and granulocytes are mainly involved in the phagocytosis of bacteria [53], fungi [54] and microsporidia [55]. Another important immunological event occurring against bacterial, fungal or viral infections is nodulation, where haemocytes become adherent in nature initially, exhibit morphological changes and tend to form aggregates [56]. Noduler protein is known to mediate the nodulation reaction after infections as it has the properties of binding to target pathogens as well as haemocytes [57]. Haemocytes exhibiting degranulation release PPO and other activators, which recruit other cells in the vicinity to initiate aggregation, nodulation or encapsulation reactions. On the basis of size of invading foreign target, the encapsulation process is activated by formation of multilayer capsule of haemocytes followed by melanisation [58].

Melanisation is an independent pathway induced in haemolymph, haemocytes and other immunocompetent tissues of *B. mori* upon bacterial, viral, fungal or parasitic infection. The melanisation reaction is initiated on the recognition of pathogens by host PRRs, which activates the serine protease cascade. The pro-PPAE (pro-prophenoloxidase activating enzyme) is converted to active PPAE via serine proteases. PPAE catalyse the conversion of PPO to active phenoloxidase (PO) enzyme. Further, PO activates the catalytic reaction of phenol-oxidation to quinones leading to the formation of insoluble melanin [59]. Serine proteases which regulate the melanisation reaction are inhibited by a family of proteins called serpins (serine protease inhibitors) found in *B. mori.* It is reported that specific serpins are induced significantly in silkworms challenged with bacterial pathogens. One such protein, serpin-6 (BmSP6) regulated the immune pathway in silkworms by inhibiting the activation of PPO and induction of an AMP, peverin-2 [60]. According to the recent findings, silkworms injected with recombinant BmSP6 and serpin-5 (BmSp5) led to the reduction in expression of AMPs gloverin3, cecropinD, cecropinE and gloverin2, respectively [61,62].

The humoral immune response is mainly activated in the fatbody and, in some instances, in other immunocompetent tissues such as integumental epithelium, midgut epithelium and haemocytes [63,64,65,66,67]. Humoral immunity includes the activation of signalling pathways such as Toll, Immunodeficiency (Imd) and Janus kinase/signal transducers and activators of transcription (JAK/STAT) pathways in immune responsive tissues and cells of hosts to produce AMPs and other effector molecules. The Toll pathway is activated upon recognition of Gram positive bacteria and fungi. Spätzle processing enzyme (SPE) activates Spätzle (Spz), which binds to the Toll receptors to initiate the assembly of TISC (Toll induced signalling complex; three members with Death Domain), in turn activating DIF (dorsal related immunity factor), the NF-κB transcription factor, which translocates to the nucleus and initiates synthesis of AMPs [59,68]. On the recognition of Gram negative bacteria, the Imd pathway induction in the host occurs through activation of a series of pathway components. The phosphorylation of Relish and its cleavage releases the active NF-κB transcription factor, REL, which binds to a distinct κB site and activates AMP synthesis [59,69]. AMPs are the most common humoral effector molecules, of which six families (namely cecropin, defensin, attacin, moricin, gloverin and lebocin) have been identified and reported in silkworms [70,71,72]. AMPs produced as the final product of immune pathways are released into the haemolymph. Another pathway, the JAK/STAT pathway is activated upon binding of Upd (unpaired) to receptor named Dome (domeless-a dimer) which initiates phosphorylation of JAK. This recruit phosphorylated STATs and binding of JAK/STAT leads to activation of gene transcription [73,74]. The activation of other cascades viz., melanisation, ROS generation and coagulation in immunocompetent tissues are immediate reactions to the invasion of foreign pathogens.

## 3. Isolation of AMPs from Silkworms

The larval stage of silkworm, *B. mori*, has five instars during which the larvae go through four moults. Silkworms are challenged with pathogens to isolate AMPs during their fifth instar as the duration is longer (6–8 days), which allows for enough time to develop infection. Furthermore, silkworm’s fat body content is at its peak during this instar, which is the primary source of AMPs [75,76]. Upon infection, the immunocompetent tissues are lysed in a suitable buffer to extract the proteins and subjected to various chromatographic techniques such as ion-exchange chromatography, gel filtration chromatography and RP (reverse phase)-HPLC (High performance liquid chromatography) for purification. Finally, the purified peptides are identified via mass spectrometry and de novo sequencing [77]. The proteomic data are analysed using the following tools: Mascot Distiller coupled with Mascot Server [78], Thermo proteome discoverer [79], PEAKS [80], Maxquant and a companion software, Perseus [81,82]. The antibacterial activity of the isolated AMPs against test cultures can be determined by employing any of the following techniques viz., agar disc diffusion, agar well diffusion, agar plug diffusion, antimicrobial gradient method, broth microdilution, broth macrodilution and agar dilution method [83].

## 4. Different AMPs in Silkworms

AMPs from silkworm are low molecular weight proteins (<50 amino acid residues; <10 kDa with few exceptions) among which a majority of them exhibit broad spectrum activity against different microorganisms. The different families of AMPs characterized and reported in silkworms, their characteristics, structure, mode of action and efficacy against bacterial species are presented in Table 2.

### 4.1. AMPs Reported from Mulberry Silkworm B. mori

#### 4.1.1. Cecropins

Cecropin, α-helical linear AMP (37 amino acids) lacking cysteine residues was first isolated from *Hyalophora cecropia* moth infected with bacteria [84]. In *B. mori*, five types of cecropins are found including cecropin A, cecropin B, cecropin C, cecropin D and cecropin E. A total of eleven *Bmcec* genes (*BmcecA1*, *BmcecA2*, *BmcecB1*, *BmcecB2*, *BmcecB3*, *BmcecB4*, *BmcecB5*, *BmcecC*, *BmcecD1*, *BmcecD2* and *BmcecE*) encoding cecropins are reported in silkworms [85]. Another AMP, enbocin, whose amino acid composition indicated that it belonged to the cecropin family, was also reported in *B. mori* [71,86]. Cecropins are primarily produced in the host mainly in response to Gram positive or Gram negative bacterial infections. They possess random coil structures in aqueous solution, but when they interact with cell membrane of microorganisms, they adopt α-helical conformations [87,88]. Although certain aspects of cecropins’ mode of action are still unknown, it is presently believed that they bind to the bacterial cell membrane along the axes of α-helical domains parallel to lipid bilayer. The polar residues of cecropins attach to the lipid phosphates, whereas the non-polar side chains burrow into the membranes hydrophobic core. The continuous accumulation of cecropin molecules forms a carpet structure on lipid bilayer surface, which has a detergent-like property and disintegrates the bacterial membrane [88,89]. However, *H. cecropia* cecropins at lower concentrations interact with membranes to form channels or pores, affecting cellular electrolyte balance, thereby causing cell death [88,90].

Cecropins at very low concentrations exhibit antibacterial activity against a wide range of Gram positive and Gram negative bacteria (Table 2). Two modified *B. mori* cecropins, CecXJ-37C and CecXJ-37N with an amino acid addition on C-terminal, are also reported to be active against diverse bacterial strains [91]. Cecropins and cecropin-type peptides are also known to inhibit growth of *Aspergillus* spp., *Fusarium* spp. and yeasts indicating antifungal properties of this AMP [92]. These peptides are shown to have low cytotoxicity and negligible haemolytic activity to the host cells at concentrations exhibiting antimicrobial activity. The ability of cecropins like any other AMP to preferentially target microbes without interfering with host cells is due to differences in the makeup and composition of the respective cell membranes [93]. Reports suggest that *B. mori* cecropins did not exhibit any cytotoxic or haemolytic effects at concentrations up to 200 µM, but they inhibited growth of microbial pathogens at much lower concentrations [87].

Apart from antimicrobial properties, cecropins are reported to selectively induce apoptosis in cancer cells. CecropinXJ, a newly isolated cationic AMP from *B*. *mori* inhibited growth of hepatocellular carcinoma (HCC) cell line Huh-7 cells and induced apoptosis in HCC cells [94]. CecropinA is also reported to induce apoptosis in human leukaemia (HL-60) cells [95]. Cecropins, like most AMPs, are known to specifically target tumour cells by binding to the phospholipid phosphatidylserines found on the outer surface of tumour cell plasma membranes. This sort of membrane architecture differs in normal cells, where phospholipid phosphatidylserines are found in the inner surface of plasma membrane and phosphatidylcholines and sphingomyelins are located on the outer surface [96].

#### 4.1.2. Defensins

Defensins are cysteine containing peptides that were first reported from *Sarcophaga peregrina*, the flesh fly [97]. Defensins are cationic in nature containing conserved cysteine residues (6 no’s) and have molecular weight of 4 kDa. Defensins have a complex structural topology with arrangement of α-helixes and β-sheets stabilized by three disulfide-bonds and therefore known as cysteine-stabilized αβ motif [98]. *BmDefensinA* found in *B. mori* genome is a defensin ortholog of *Spodoptericin*. A group of researchers reported expression of *BmDefensinB* gene in *B. mori* after infection with *E. coli*, *Bacillus subtilis* and *Beauveria bassiana* [99]. Defensins exhibit antibacterial activity against Gram-positive bacteria, namely *B. subtilis*, *B. thuringiensis*, *B. megaterium Micrococcus luteus*, *S. aureus* and *Aerococcus viridians* [23]. A defensin-like anionic antimicrobial peptide BmDp from *B. mori* is also reported, which is identical to BmDefensinA and is close to galiomicin and spodoptericin [100].

Defensins inhibit bacterial growth by membrane disruption and through the formation of voltage dependent anion-selective channels in cell membranes [101,102,103]. Recent findings suggest that β-defensin binds to specific phospholipids on the cell membrane forming oligomeric complex to facilitate cell lysis [104]. However, insect defensin’s mode of action appears to be complex and information on the same is limited. Specific targets for insect defensins are yet to be found, and structure-activity studies may aid in unravelling the molecular process behind their bioactivity [98].

#### 4.1.3. Moricins

Moricin, a cationic, amphipathic α-helix AMP shows the presence of charged amino acid residues after every three to four amino acids, which is responsible for its antimicrobial properties against bacteria and few strains of yeasts. Moricin consists of 42 amino acid residues and was first isolated from the *B. mori* haemolymph. It was found to be active against Gram positive bacteria *S. aureus* [77]. In *B. mori*, a total of twelve genes encoding moricin have been reported and divided into two subfamilies on the basis of sequence similarity. Out of twelve moricin genes, four belong to subfamily *BmmorA* (A1 to A4) and eight belong to subfamily *BmmorB* (B1 to B8) [85].

A very limited amount of literature is available on the mechanism of pore formation in bacterial cell membrane by moricins from *B. mori*. Moricin contains N-terminal fragment (5–22 amino acids), which is amphipathic, α-helical and is the active site for antibacterial activity. The C-terminal region of moricin initially interacts with the surface of bacterial membrane and then permeability of the membrane is altered by N-terminal amphipathic a-helix. It is reported that the voltage-dependent pores could be formed through interaction between three or more amphipathic α-helices spanning a lipid membrane [77,105]. Moricins exhibit higher antibacterial activity against Gram positive bacteria than Gram negative bacteria.

#### 4.1.4. Gloverins

Gloverins are glycine-rich AMPs of molecular weight 8–30 kDa and were first reported from haemolymph of giant silk moth *H. gloveri* pupae [106]. Gloverins possess flexible random-coil structure in aqueous solution. Gloverins from different insects are active against bacteria, fungi and virus while inactive against *E. coli* strains possessing smooth LPS. Reports suggest that the binding of gloverins to the inner part and Lipid A region of LPS is required for its activity. A conformational change occurs in the gloverins when they penetrate the hydrophilic regions of LPS layer and interacts with negatively charged hydrophobic regions made of lipid A [106,107]. BmGlvs binds to rough LPS leading to conformational transition of this peptide from random coil to α-helix which is believed to be the main reason for pore formation on bacterial cell membrane [107]. Binding of gloverin to microbial surface is prerequisite for its conformational change and antimicrobial activity.

In silkworm *B. mori*, four genes encoding gloverins (namely *Bmglv1*, *Bmglv2*, *Bmglv3* and *Bmglv4*) were identified. All four gloverin genes were activated by *E. coli*, *B. subtilis*, and *Salmonella abortusequi* while the expression of gloverin genes was reduced when challenged with *S. aureus* [71]. The differences in the structure and compositions of bacterial cell wall among the bacterial strains may be reason for differential expression of gloverins [71]. BmGloverin2 (BmGlv2), along with other AMPs of silkworm, is reported to have synergistic antifungal activity against *B. bassiana* [108]. It is also reported that BmGLv2 inhibited growth of two Gram negative bacteria (*E. coli* JM109 and *Pseudomonas putida*) by enhancing the cell membrane permeability [109], resulting in disruption of the ion gradient between cytoplasm and external milieu and leading to cell death.

#### 4.1.5. Attacins

Attacins are low molecular weight (20–23 kDa) AMPs that were first isolated from the haemolymph of *H. cecropia* pupae inoculated with bacteria [110]. On the basis of isoelectric points (pI: 5.7–8.3), attacins are divided into two groups, namely acidic (E and F) and basic (A to D). Attacin-A1 is reported to possess antimicrobial activity against *E. coli* and *Trypanosoma brucei* [111], whereas attacin-B has antibacterial activity against Gram negative bacteria (*E. coli* and *Citrobacter freundii*) and also antifungal activity (*C. albicans*) [112]. Attacins inhibit the bacterial growth by hindering the synthesis of outer cell membrane proteins viz., OmpC, OmpF, OmpA and LamB in bacteria or by altering the permeability of bacterial outer membrane [113,114].

#### 4.1.6. Lebocins

Lebocins (32 amino acids) are proline-rich AMPs with O-glycosylated residues that were isolated from *B. mori* haemolymph challenged with *E. coli*. Lebocin family consists of four protein encoding genes (*Leb1*, *Leb2*, *Leb3* and *Leb4)*. The expression of lebocin genes is induced by LPS in haemocytes and fatbody [115]. Lebocin is reported to exhibit antimicrobial activity against Gram negative (*Acinetobacter* sp. and *E. coli*), Gram positive bacteria and fungi. Lebocin-B and Lebocin-C isolated from another lepidopteran insect, *Manduca sexta*, differ from *B. mori* Lebocin and are reported to possess antibacterial activity against *Serratia marcescens*, *S. typhimurium* (Gram negative); *S. aureus*, *B. cereus* (Gram positive) and *Cryptococcus neoformans* (fungi) [116].

**Table 2:** Antimicrobial peptides reported in silkworm, *Bombyx mori* and their antibacterial activity.

AMP	Characteristics	Structure	Mode of action	Microorganisms	MIC	LC	Ref.
Cecropins	Cationic	α-helix	Pore forming; Forms a carpet structure on lipid bilayer surface and disintegrates the bacterial membrane				
BmcecA1				*B. subtilis*	2.5 µM		[117]
				*B. thuringiensis*	2.5 µM		
				*E. coli*	2.5 µM		
				*P. aeruginosa*	2.5 µM		
				*Ralstonia dolaanacearum*	2.5 µM		
Cecropin B1				*P. fluorescens*		1.6 µM	[118]
				*Xanthomonas campestris*		1.2 µM	
				*Chromobacterium iodinum*		0.85 µM	
				*Agrobacterium tumefaciens*		0.41 µM	
				*Alcaligenes faecalis*		0.49 µM	
				*Achromobacter polymorph*		1.8 µM	
				*E. coli* K12		0.38 µM	
				*S. marcescens*		0.67 µM	
				*M. luteus*		0.59 µM	
				*S. aureus*		>10 µM	
				*Brevibacterium ammoniagenes*		0.49 µM	
				*Lactobacillus plantarum*		0.62 µM	
				*Arthrobacter simplex*		0.46 µM	
				*B. subtilis*		3.6 µM	
				*B. sphaericus*		4.4 µM	
Cecropin B				*B. megaterium*		1.7 µM	[89]
				*E. coli*		0.35 µM	
				*M. luteus*		>207 µM	
				*P. aeruginosa*		10 µM	
				*S. marcescens*		17.22 µM	
BmcecB6				*B. bombysepticus*	2.5 µM		[117]
				*B. subtilis*	2.5 µM		
				*B. thuringiensis*	1.25 µM		
				*B. thuringiensis* subsp. *galleriae*	1.25 µM		
				*E. coli*, *S. marcescens*, *P. aeruginosa*	0.625 µM		
				*R. dolaanacearum*	1.25 µM		
BmcecD				*B. bombysepticus*	2.5 µM		[117]
				*B. subtilis*	2.5 µM		
				*B. thuringiensis*	1.25 µM		
				*B. thuringiensis* subsp. *galleriae*	2.5 µM		
				*E. coli*, *P. aeruginosa*, *R. dolaanacearum*	1.25 µM		
				*S. marcescens*	2.5 µM		
BmcecE				*B. thuringiensis*	1.25 µM		[117]
				*E. coli*, *S. marcescens*, *P. aeruginosa*, *R. dolaanacearum*	2.5 µM		
Modified Cecropins CecXJ-37C				*E. coli* ATCC 25922	3.9 µM		[91]
				*P. aeruginosa* ATCC 27853	3.9 µM		
				*K. pneumoniae* ATCC 700603	15.7 µM		
				*S. aureus* ATCC 25923	0.25 µM		
				*S. aureus* ATCC 29213	1 µg/mL		
				*S. aureus* ATCC 43300	1 µg/mL		
				*B. subtilis* ATCC 6633	1 µg/mL		
				*S. epidermidis* ATCC 12228	1 µg/mL		
CecXJ-37N				*E. coli* ATCC 25922	1 µM		[91]
				*P. aeruginosa* ATCC 27853	1 µM		
				*K. pneumoniae* ATCC 700603	7.8 µM		
				*S. aureus* ATCC 25923	0.25 µM		
				*S. aureus* ATCC 29213	1 µg/mL		
				*S. aureus* ATCC 43300	1 µg/mL		
				*B. subtilis* ATCC 6633	1 µg/mL		
				*S. epidermidis* ATCC 12228	1 µg/mL		
Defensins	Cationic Cysteine rich Hydrophilic peptide	Cysteine-stabilized αβ motif	Disrupts bacterial cell membrane; Formation of voltage dependent anion-selective channels in cell membranes	*S. aureus*	NP		[101,102,103]
Moricins	Basic Amphipathic	α-helix	Alters the membrane permeability; Formation of voltage dependent pores	*E. coli* JM 109		0.31 µM	[77,105]
				*Acinetobacter* sp. NISL B-4653		0.27 µM	
				*P. fluorescens* IAM 1179		0.53 µM	
				*P. aeruginosa* IAM 15140		0.81 µM	
				*B. subtilis* IAM 1107		0.19 µM	
				*B. megaterium* IAM 1030		0.09 µM	
				*B. cereus* IFO 3457		0.38 µM	
				*S. aureus* ATCC 6538P		0.21 µM	
				*S. aureus* ATCC 6538Pa		0.22 µM	
				*S. aureus* IFO 3083		0.46 µM	
				*S. xylosus* IAM 1312		0.27 µM	
				*S. epidermidis* IFO 12993		0.18 µM	
				*S. pyogenes* ATCC 21547		0.25 µM	
Bmmor				*S. aureus*, *B. subtilis*	0.625 µM		[117]
				*B. bombysepticus*, *B. thuringiensis*, *B. thuringiensis* subsp. *galleriae*, *E. coli*, *P. aeruginosa*, *R. dolaanacearum*	1.25 µM		
				*S. marcescens*	0.625 µM		
Gloverins	Glycine rich Acidic to neutral (pI: 5.5–7.2)	Random coil	Pore forming				[117]
Bmglv1				*B. thuringiensis*	1.4 µM		
				*B. thuringiensis* subsp. *galleriae*	1.6 µM		
				*E. coli*, *P. aeruginosa*, *R. dolaanacearum*	1.4 µM		
				*S. marcescens*	1.2 µM		
				*X. campestris*	1.6 µM		
Bmglv2				*B. thuringiensis*, *B. thuringiensis* subsp. *galleriae*, *E. coli*, *S. marcescens*, *R. dolaanacearum*	1.6 µM		
				*X. campestris*, *P. aeruginosa*	1.8 µM		
Bmglv3				*B. thuringiensis*, *B. thuringiensis* subsp. *galleriae*, *S. marcescens*, *R. dolaanacearum*	1.6 µM		
				*E. coli*	1.4 µM		
				*P. aeruginosa*, *X. campestris*	1.8 µM		
Bmglv4				*B. thuringiensis*, *E. coli*, *S. marcescens*, *R. dolaanacearum*	1.4 µM		
				*B. thuringiensis* subsp. galleriae, *P. Aeruginosa*, *X. campestris*	1.6 µM		
Attacins	Glycine-rich	Random coil structure in aqueous solution	Altering cell membrane permeability; Hampers synthesis of plasma membrane proteins of bacterial cell	Gram negative and Gram positive bacteria	NP		[119]

MIC—Minimum inhibitory concentration; LC—Lethal concentration; NP: Data not provided.

### 4.2. AMPs Reported from Non-Mulberry Silkworms

In addition to the AMPs from the domesticated mulberry silkworm, *B. mori*, AMPs have also been identified from the nonmulberry silkworms belonging to the family Saturniidae, namely *Antheraea assamensis* (muga), *Antheraea mylitta* (tropical oak tasar), *Antheraea pernyi* (temperate oak tasar), *Antheraea yamamai* (Japanese oak tasar) and *Samia cynthia ricini* (eri).

An antifungal peptide named gallerimycin is reported to be isolated from the fatbody of *S. cynthia ricini*. A cDNA clone of *Scr-gallerimycin* (AB366558) gene encodes 74 amino acids and the gallerimycin protein has 6.21 kDa of calculated molecular mass and 7.6 pKa [120]. A lebocin-like gene induced in the fatbody of eri silkworms upon challenging with bacteria was also reported. The cDNA of the lebocin-like gene encodes for 162 amino acids, which has homology with *B. mori* and *Trichoplusia ni* lebocin precursor proteins [121]. The cDNA clones of two Attacins (A and B) were reported from the fatbody of *S. cynthia ricini* challenged with bacteria. Both the attacin genes were coded for 233 amino acids and shared 98% identity at protein level, whereas at nucleotide level, 92% identity was reported [122]. Another attacin-like gene was reported from *A. pernyi* whose expression level significantly increased in fatbody upon *E. coli* infection [123]. A gloverin-like peptide of molecular mass 9.052 kDa active against Gram negative bacteria was isolated and characterized from muga silkworm immunized with *C*. *albicans* [124]. In *A. mylitta*, a glycine-rich antimicrobial peptide (GGGGGGHLVA) was reported to be active against MDR *E. coli* associated with urinary tract infections [125]. A tri-peptide AMP, NH_2_-Gln-Ala-Lys-COOH (QAK) was reported to be isolated and purified from haemolymph collected from immunized *A. mylitta*. Acetylated and non-acetylated QAK peptide exhibited antibacterial activity against *E. coli* and *S. aureus* [126]. A cecropin-like peptide isolated from the Japanese oak silkworm, *A. yamamai* exhibited antimicrobial activity against Gram negative bacteria (*E. coli*, *K. pneumoniae* and *P. aeruginosa*), Gram positive bacteria (*S. aureus*, *Enterococcus faecalis* and *M. luteus*) and fungi (*C. albicans*), indicating its broad spectrum potential. The authors reported that MIC values against the tested Gram negative bacteria, Gram positive bacteria and fungal strain ranged between 1–2 µg/mL, 64–128 µg/mL and 64 µg/mL, respectively [127]. In another study, AMPs were isolated from haemolymph samples of *A. mylitta* and fractionated by HPLC. The fractions were assessed for their antibacterial activity against MDR bacteria including *E. coli*, *P. aeruginosa* and *B. pumilus.* It was found that fraction II exhibited antibacterial activity against *E. coli* (zone of inhibtion-9 ± 0.35 mm) and *P. aeruginosa* (6.5 ± 0.40 mm), whereas fraction III was active against only *B. pumilus* (7.5 ± 0.30 mm) [128].

## 5. Factors Affecting the Activity of AMPs

The AMPs isolated from natural sources are generally unstable, and it is therefore imperative to determine their stability before going ahead with application in various fields. AMPs are affected by several factors such as metal ions, temperature, pH and proteases. Metal ions affect the self-assembly and activity of AMPs, while pH may have varied effects depending upon the charge of the peptides [3]. The majority of the AMPs show poor stability at ambient temperatures. The stability of peptide is determined at different temperatures ranging from 4 °C to 90 °C incubated for minutes to days depending upon the application of AMP [129,130]. Upon incubation, AMPs are again evaluated for antimicrobial activity via the microdilution well method to determine the minimum inhibitory concentration (MIC). In the case of some AMPs, MIC increased with incubation time, while a few reports suggested that AMPs are stable even at higher temperatures and longer incubation times [129,130]. Proteases exert a highly destructive effect on AMPs. The effect of proteases on AMPs is assessed by exposure of the AMPs to proteinase K, chymotrypsin and trypsin. All three proteases are known to reduce the antimicrobial activity of AMPs as they act by degradation of AMP or by inhibition of the AMP activity [129,131].

In order to overcome the influence of different factors mentioned above, the identified bioactive peptides could be synthesized chemically through solid-phase peptide and peptide synthesis in solution. Chemical synthesis of AMPs is advantageous over extraction of AMP from natural sources, as synthetic peptides are easy to modify as per the specific requirement [132,133]. More efficient analogues of AMPs may be prepared with better activity and stability. The stability of AMPs against proteases is reported to be improved by different chemical modifications such as capping (acetylation or amidation of residues), residue phosphorylation, cyclization, the addition of unnatural amino acids or D-amino acids and peptidomimetics [3]. In view of these reasons, designed AMPs have attracted many researchers for obtaining the desired effects. During the designing of AMP, the length, net charge, secondary structure, hydrophobic and amphiphilic properties of the peptide have to be considered to ensure its bioactivity. Additionally, the conjugation of fatty acid to side chain of peptide helps in improvement of stability, antibacterial selectivity and antibiofilm activity of AMPs [3,134].

## 6. Current Status: AMPs under Clinical Investigation

The most recent AMP data collected from the DRAMP (Data repository of antimicrobial peptides) database (http://dramp.cpu-bioinfor.org/; accessed on 27 April 2023) indicate 22,480 submissions, including natural and synthetic AMPs (6105 No’s), patent AMPs (16,110 No’s) and 77 AMPs in preclinical or clinical stages of drug development. Forty three percent of the 77 AMPs are in the preclinical stage and 33 peptides are in clinical trials. Six peptides failed during phase III studies, while one was denied permission [135]. Although some AMPs have demonstrated therapeutic efficacy in vitro or in vivo, the majority of them have failed clinical trials due to a variety of difficulties [136]. In the recent decades, the FDA granted approval for two antimicrobial lipopeptides, daptomycin and oritavancin. Daptomycin is produced by *Streptomyces roseosporus* and is employed in the treatment of complicated skin and skin structure infections. Daptomycin exhibited vast antibacterial activity against methicillin-susceptible and methicillin-resistant *S*. *aureus*, Vancomycin susceptible *Enterococcus faecalis* and different *Streptococcus* species [137]. Oritavancin is a lipoglycopeptide used to treat adults with acute bacterial skin and skin structure infections caused by microorganisms including methicillin-susceptible and methicillin-resistant *S*. *aureus*, different strains belonging to the genus *Streptococcus* and *E. faecalis* [138].

## 7. Conclusions

The antibiotic resistance crisis has led to exploration for novel therapeutics globally. AMPs have the potential to be an effective treatment method against drug-resistant bacteria. A variety of animals, including insects, that manufacture AMPs as a component of their innate immune system are being studied for novel AMPs, and silkworm is one of them. The silkworm has one of the most extensive repertoires of structurally and functionally distinct AMPs with antimicrobial activity against different microorganisms. This study draws attention to silkworm as a possible source of various antimicrobial peptides and opens the door to new avenues for intervention and the development of naturally occurring bioactive compounds to address antibiotic resistance.

## 8. Future Perspectives/Challenges

Like many of the insect AMPs, silkworm AMPs also have limitations in terms of low bioavailability, possible haemolysis, susceptibility to proteases, cytotoxicity, high production costs and low expression which continues to restrict their usage in clinical applications. Even though it is essential to continue exploring for novel silkworm AMPs, more research is also required to overcome the limitations preventing their clinical applications. Emphasis should also be placed on the development of transgenic or genome edited silkworms for over expression of AMPs. However, before using the AMPs for different applications, safety must be ensured. Strategies may also be employed to design better synthetic AMPs based on the sequences of AMPs from natural sources using in silico approaches.

## Figures and Tables

**Table 1:** Major diseases of silkworm, their causal microorganisms and symptoms.

Silkworm Disease	Causative Microorganism/s	Symptoms
Bacterial flacherie	▪ *Bacillus* sp. ▪ *Streptococcus* sp. ▪ *Staphylococcus* sp.	✓ Loss of appetite and stunted growth ✓ Vomiting of gut juices ✓ Translucent cephalothoracic region ✓ Rupture of skin and discharge of a dark-brown fluid with unpleasant odour ✓ Diseased larvae appear black in color
Muscardine	▪ *Beauveria bassiana* ▪ *Aspergillus flavus* ▪ *Nomuraea rileyi*	✓ Impairment in elastic properties of skin ✓ Larvae upon death become hard and mummified ✓ Depending on the causative agent, the mummified larvae appear white or green or brown coloured
Grasserie	▪ *Bombyx mori* nuclear polyhedrosis virus	✓ Fragile integument that easily ruptures and exudes white haemolymph ✓ Swollen intersegmental regions ✓ Diseased larvae crawls continuously across the edges of the rearing tray
Pebrine	▪ *Nosema bombycis*	✓ Only transovarially transmitted silkworm disease ✓ Uneven/delayed moulting ✓ Retardation in growth ✓ Larvae belonging to same developmental stage exhibit variations in size

## Data Availability

Not applicable.
